# Shigella flexneri Bacteremia in an Immunocompetent Woman With Socioeconomic Risk Factors: A Case Report

**DOI:** 10.7759/cureus.82648

**Published:** 2025-04-20

**Authors:** Aabid Mohiuddin, Fawaz Hussain, Ayesha A Arabi, Abdallah Almawazreh, Saumya L Karne, Wael Dahhan

**Affiliations:** 1 Department of Internal Medicine, Detroit Medical Center/Wayne State University, Detroit, USA; 2 Department of Health Sciences, University of Sharjah, Sharjah, ARE; 3 Department of Gastroenterology, Wayne State University School of Medicine, Detroit, USA

**Keywords:** immunocompetent, infectious diarrhea, shigella, shigellemia, shigellosis

## Abstract

*Shigella* species are responsible for millions of cases of shigellosis each year worldwide, particularly in underdeveloped countries. These bacteria invade the colonic epithelium and release enterotoxins, leading to watery or bloody diarrhea, fever, and abdominal pain. Bacteremia is a rare systemic complication of shigellosis, with only a few cases reported among immunocompetent adults. Here, we present an intriguing case of *Shigella* flexneri bacteremia in an elderly, immunocompetent woman. Uniquely, the patient’s stool tested negative for Shiga toxin despite confirmed shigellemia. In addition to its unusual clinical presentation, this case offers important epidemiologic insights. It underscores the need for clinicians to consider patients’ socioeconomic backgrounds when forming a broad differential diagnosis and to remain vigilant for diseases that are less commonly encountered in resource-rich settings.

## Introduction

Shigellosis is an infectious syndrome characterized by fever, abdominal cramps, tenesmus, and bloody stools, resulting from the invasion of the colonic epithelium by *Shigella* species and the release of potent enterotoxins. Approximately 188 million people are infected globally each year, with the majority of cases occurring in young children and infants from impoverished communities and resource-limited settings, primarily through the ingestion of contaminated food and water [1]. Among the *Shigella *species, *Shigella sonnei* typically causes a self-limiting illness marked by watery diarrhea, whereas *Shigella flexneri *and *Shigella dysenteriae *are associated with more severe symptoms, including profuse vomiting, abdominal cramping, and bloody diarrhea in 35-55% of cases [2]. Systemic complications are exceedingly rare and are typically observed in immunocompromised individuals or those with underlying conditions such as diabetes mellitus [3]. This case highlights a rare presentation of *S. flexneri *bacteremia in an elderly, immunocompetent woman residing in the United States.

## Case presentation

A 66-year-old immunocompetent woman with a history of paroxysmal atrial fibrillation, congestive heart failure, and well-controlled diabetes mellitus was admitted to the medicine service with complaints of fatigue, mild abdominal cramping, and diarrhea lasting for the past week. She was considered relatively immunocompetent in the absence of any overt or uncontrolled systemic disease. She reported experiencing up to five episodes of watery stools per day, occasionally streaked with bright red blood. She also noted minimal oral intake over the previous week due to nausea and abdominal discomfort.

On physical examination, she had dry oral mucosa and decreased skin turgor, consistent with dehydration. Her abdomen was soft and nondistended, with mild, diffuse tenderness on palpation. The remainder of the examination was unremarkable. No imaging was obtained upon admission. Laboratory evaluation revealed an elevated creatinine level above her baseline, likely secondary to dehydration, which improved following fluid resuscitation. There was no leukocytosis, and her hemoglobin level on admission was within the normal range at 13.6 g/dL, with a normocytic mean corpuscular volume (Table 1). She had no prior record of endoscopy.

**Table 1:** Laboratory values at admission

Parameter	Patient value	Reference range
Sodium	141 mmol/L	136-145 mmol/L
Potassium	4 mmol/L	3.6-5.1 mmol/L
Chloride	107 mmol/L	98-107 mmol/L
Bicarbonate	21 mmol/L	22-29 mmol/L
Glucose	122 mg/dL	74-109 mg/dL
Creatinine	3.34 mg/dL	0.5-1.2 mg/dL
Alanine aminotransferase	52 units/L	10-50 units/L
Aspartate aminotransferase	63 units/L	5-40 units/L
Alkaline phosphatase	105 units/L	35-129 units/L
Total bilirubin	2 mg/dL	0.15-1.2 mg/dL
White blood cell count	7.7 units/uL	1,000 units/uL
Absolute neutrophil count	4.6 units/uL	1.6-7.1 units/uL
Absolute neutrophil percentage	69%	38-76%
Hemoglobin	13.6 g/dL	11.5-15.1 g/dL
Mean corpuscular volume	99 fL	81-98 fL

**Figure 1:**
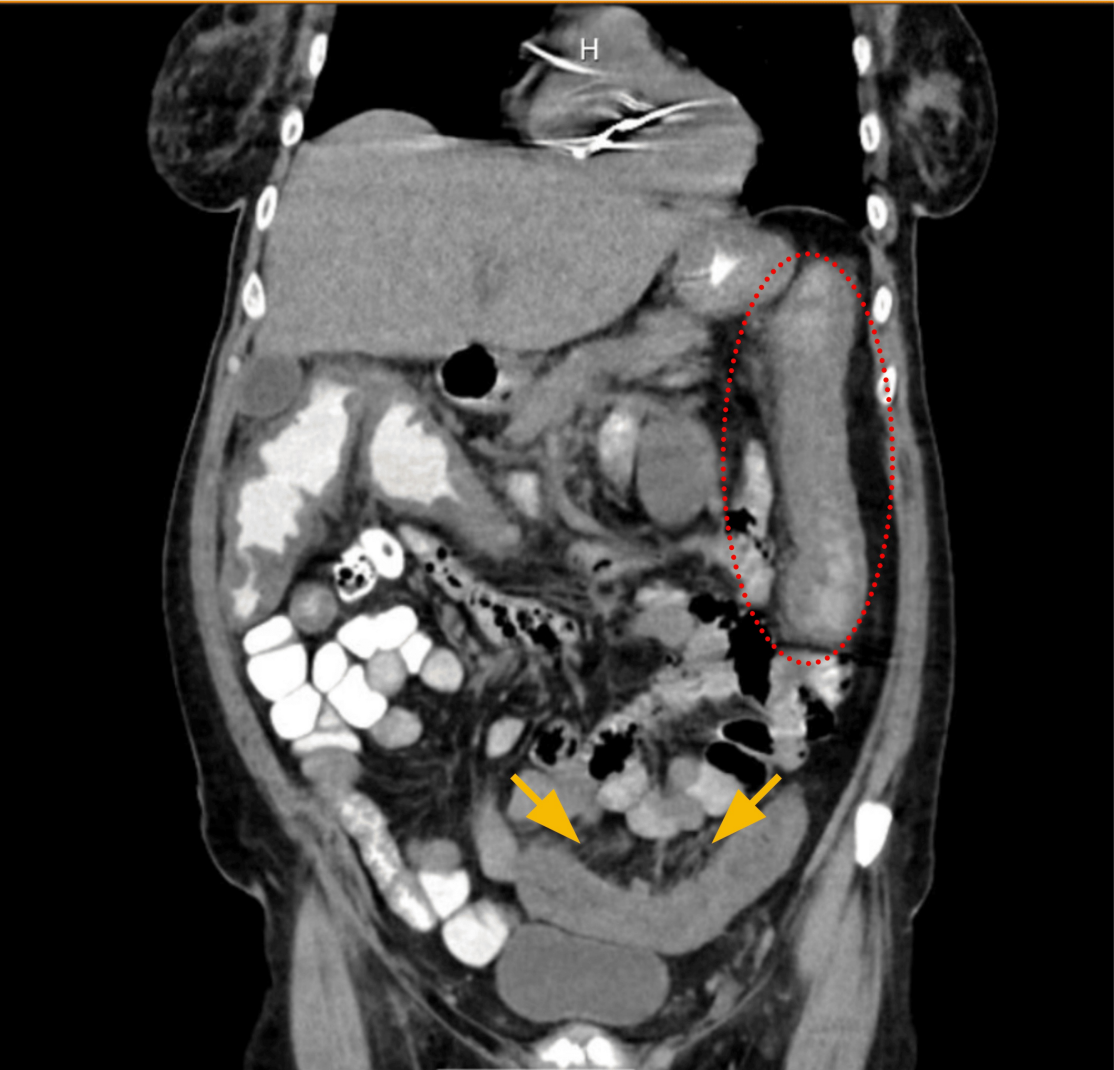
CT of the abdomen shows diffuse colonic wall thickening and peri-colonic fat stranding Yellow arrows indicate areas of peri-colonic fat stranding, while the red dotted circle highlights colonic thickening in the descending colon.

Bowel movements observed in the emergency department were watery and brown, without visible blood; however, upon admission, the patient developed acute bloody diarrhea accompanied by a significant drop in hemoglobin to 11.3 g/dL. At that time, further discussion revealed that she lived in an apartment with her daughter and her grandchildren, including an infant granddaughter who had recently experienced an episode of bloody diarrhea. An infectious diarrheal workup was initiated. Polymerase chain reaction testing for *Clostridioides difficile *was negative, and stool cultures were also negative for *Salmonella*, *Shigella*, *Campylobacter*, and ova and parasites. A Shiga toxin immunochromatographic rapid test returned negative as well. Given the negative infectious workup, a CT scan of the abdomen and pelvis was obtained, which demonstrated diffuse wall thickening throughout the colon with associated peri-colonic fat stranding, findings suggestive of an acute infectious process (Figure 1).

Given the negative bacterial infectious workup, the leading differential diagnosis at that time was viral gastroenteritis. As a result, loperamide was initiated to help alleviate her symptoms. Although her diarrhea improved with loperamide, she became febrile overnight with a temperature of 39.7°C. Blood cultures were drawn, and a preliminary result the following morning was positive for gram-negative bacilli. Aztreonam was started empirically due to the patient’s documented allergies to beta-lactam antibiotics. The initial presumed source of bacteremia was *Escherichia coli *translocation from the gastrointestinal tract secondary to her diarrheal illness.

After five days, final blood culture results revealed growth of* S. flexneri*. Despite the unexpected identification, treatment remained unchanged based on antibiotic susceptibilities. The final diagnosis was *Shigella *bacteremia complicating shigellosis. The patient was discharged home after completing her antibiotic course and showing symptomatic improvement.

## Discussion

*Shigella *bacteremia, or shigellemia, is an extremely rare occurrence, particularly in immunocompetent adults. Martin et al. reported that 87% of all shigellemia cases between 1963 and 1981 occurred in patients under 16 years of age [4]. In another study involving 11,262 cases of *Shigella *infection in the greater Atlanta, GA area, only 72 patients (0.64%) had positive blood cultures [5]. Among those with shigellemia, 51% were immunocompromised. A separate review of 22 adult shigellemia cases found that more than half had a chronic illness, most commonly diabetes mellitus [3].

This case describes a rare clinical presentation of shigellemia in an immunocompetent adult, characterized by negative stool cultures and positive blood cultures. Morduchowicz et al. found that shigellemia without positive stool cultures was associated with a higher mortality rate compared to cases where *Shigella* was detected in both blood and stool. They further recommended that in elderly or immunocompromised patients presenting with acute febrile gastroenteritis, both blood and stool cultures should be obtained to rule out shigellemia [3].

Although there are approximately 188 million annual cases of *Shigella* infection worldwide, the United States sees only an estimated 500,000 suspected cases and 15,000 laboratory-confirmed cases each year [1,6]. Thus, it is relatively uncommon for a person living in the United States to acquire a *Shigella *infection. However, this case highlights a key epidemiologic insight: clinicians must consider a patient’s socioeconomic status when evaluating diseases typically associated with resource-poor countries. A nationwide retrospective study analyzing data from 2004 to 2014 found that census tract poverty was associated with a shigellosis incidence rate ratio of 3.6 and 2.3 after adjusting for demographic factors [7]. Additionally, a 1962 study identified a strong correlation between household crowding and shigellosis incidence [8].

Notably, our patient resides in a highly underserved and impoverished neighborhood within a major urban center. She also reported living with her daughter and several grandchildren in a single apartment, suggesting a high likelihood of household congestion. These socioeconomic risk factors are critical to understanding her clinical presentation and should be taken into account when diagnosing and managing cases of shigellemia.

## Conclusions

Clinicians in resource-rich countries often overlook certain infectious diseases due to their strong association with resource-poor regions. This case challenges that assumption and underscores the importance of considering shigellosis as a potential cause of infectious diarrhea, particularly in individuals from low socioeconomic backgrounds, who remain at increased risk despite the overall low national prevalence. Moreover, clinicians should maintain a heightened index of suspicion for *Shigella *bacteremia when evaluating elderly or immunocompromised patients presenting with acute febrile gastroenteritis - even in the absence of positive stool cultures. Prompt collection of both blood and stool cultures is essential to guide appropriate treatment and reduce the risk of complications.
